# Impact of Capsular Switch on Invasive Pneumococcal Disease Incidence in a Vaccinated Population

**DOI:** 10.1371/journal.pone.0003244

**Published:** 2008-09-19

**Authors:** Laura Temime, Pierre-Yves Boelle, Lulla Opatowski, Didier Guillemot

**Affiliations:** 1 CNAM, Paris, France; 2 INSERM UMR-S707, Paris, France; 3 Université Pierre et Marie Curie, Paris, France; 4 Institut Pasteur, PhEMI, Paris, France; 5 INSERM, U 657, Paris, France; 6 Faculté de Médecine Paris Ile de France Ouest, Université Versailles Saint Quentin, Versailles, France; Columbia University, United States of America

## Abstract

**Background:**

Despite the dramatic decline in the incidence of invasive pneumococcal disease (IPD) observed since the introduction of conjugate vaccination, it is feared that several factors may undermine the future effectiveness of the vaccines. In particular, pathogenic pneumococci may switch their capsular types and evade vaccine-conferred immunity.

**Methodology/Principal Findings:**

Here, we first review the literature and summarize the available epidemiological data on capsular switch for *S. pneumoniae*. We estimate the weekly probability that a persistently carried strain may switch its capsule from four studies, totalling 516 children and 6 years of follow-up, at 1.5×10^−3^/week [4.6×10^−5^–4.8×10^−3^/week]. There is not enough power to assess an increase in this frequency in vaccinated individuals. Then, we use a mathematical model of pneumococcal transmission to quantify the impact of capsular switch on the incidence of IPD in a vaccinated population. In this model, we investigate a wide range of values for the frequency of vaccine-selected capsular switch. Predictions show that, with vaccine-independent switching only, IPD incidence in children should be down by 48% 5 years after the introduction of the vaccine with high coverage. Introducing vaccine-selected capsular switch at a frequency up to 0.01/week shows little effect on this decrease; yearly, at most 3 excess cases of IPD per 10^6^ children might occur due to switched pneumococcal strains.

**Conclusions:**

Based on all available data and model predictions, the existence of capsular switch by itself should not impact significantly the efficacy of pneumococcal conjugate vaccination on IPD incidence. This optimistic result should be tempered by the fact that the selective pressure induced by the vaccine is currently increasing along with vaccine coverage worldwide; continued surveillance of pneumococcal populations remains of the utmost importance, in particular during clinical trials of the new conjugate vaccines.

## Introduction

Pneumococcal disease is a major cause of mortality and morbidity worldwide. In the United States alone, *S. pneumoniae* was responsible for 3 000 cases of meningitis, 500 000 cases of pneumonia, and 7 000 000 cases of otitis media each year before the introduction of conjugate vaccination in 2000 [1].

A heptavalent pneumococcal conjugate vaccine has been available for a few years. This vaccine covers the 7 most frequently carried pneumococcal serotypes in the United States, which are also among the most invasive serotypes. Since its introduction, a marked decrease in the incidence of invasive pneumococcal disease (IPD) has been reported [2]. Because of herd immunity, this decrease is not restricted to vaccinated children and has been observed in the entire population [2], [3].

Nevertheless, several factors may undermine the efficacy of the vaccine. Several studies have reported evidence of replacement of vaccine serotypes by non-vaccine serotypes in vaccinated populations [4], [5]. Mathematical models have suggested that this may in the long term cancel the impact of vaccination on pneumococcal colonization rates [6], [7] and lessen its impact on IPD incidence [8].

Another worrying factor is the suspicion that some highly invasive or antibiotic resistant pneumococci may have a propensity to switch their capsular type, thereby evading vaccine-conferred immunity [9]–[11]. Several studies have indeed reported the emergence in vaccinated populations of new pneumococcal clones expressing non-vaccine serotypes, but genetically closely related to vaccine serotypes [9], [11]–[14]. In particular, since the introduction of conjugate vaccination, serotype 19A has emerged as the predominant invasive pneumococcal serotype in the United States [15]. Molecular epidemiology analyses suggest that this increase is largely attributable to the expansion of capsular switch variants [16]. However, evidence of capsular switch has also been reported in *S. pneumoniae* before the vaccination era, and the influence of the conjugate vaccine remains unclear [9], [17], [18]. Furthermore, little is known on the actual frequency at which pneumococci may switch their capsular type [18], [19].

In this study, we first review 4 studies presenting longitudinal data on pneumococcal carriage in the population to estimate the frequency of “natural” vaccine-independent capsular switch. Since it is suspected that conjugate vaccination may favour selection of capsular switch in vaccine-covered or related serotypes [9], [14], we specifically investigate this possibility from those of the studies which also provide data on the vaccination status of individuals.

Secondly, we use the gathered information in a mathematical model of pneumococcal transmission to quantify the possible changes induced by capsular switch on the incidence of invasive pneumococcal disease in vaccinated populations.

## Methods

### Review on pneumococcal capsular switch

In this article, we distinguish between “natural” capsular switch, which is the propensity for a pneumococcal strain to switch its capsular type independently of its environment, and vaccine-selected capsular switch, which results from the selective pressure of conjugate vaccination on pneumococcal strains. The vaccine-selected capsular switch phenomenon is added to the natural capsular switch phenomenon in vaccinated individuals.

#### Search strategy and selection criteria for the review

Data for the review were identified by a systematic search of Medline and references from relevant articles. Search terms were “longitudinal” (or “birth”, “scheduled”, “month*”, “week*”, “age*”), “surveillance” (or “cohort”, “trial”, “monitor*”, follow*”), “colonization” (or “carriage”), “isolate*” (or “sample*”), “serotype*” (or “genetic*”, “multilocus”, “capsul*”, “molecular”) and “pneumoniae” (or “pneumococc*”).

English and French language papers were reviewed. We included only those studies which provided detailed longitudinal data on pneumococcal carriage in a population with information on serotypes and MLST genotypes of carried isolates at all sampling times, as well as reports on observed capsular switches. Conversely, we excluded studies which did not provide detailed enough data.

#### Frequency of vaccine-independent “natural” capsular switch

Capsular switch events were defined in all studies as the consecutive isolation in persistently colonized individuals of two isolates that were identified by MLST as closely related, but that expressed different serotypes. The frequency of capsular switch in a study was estimated as the ratio of the number of observed switches by the total time during which switches may have occurred.

In order to obtain the denominator, which represented the time at risk for capsular switch, we computed the duration of observed persistent pneumococcal colonization for all children included in the study. For any given child, this was defined as the sum of all durations between 2 consecutive positive samplings in the course of the study. The time at risk was then calculated as the total duration of persistent pneumococcal colonization, cumulated over all children.

#### Investigation of vaccine-selected capsular switch

Vaccine-selected capsular switch events were defined as the consecutive isolation in persistently colonized vaccinated individuals of a vaccine and a non-vaccine isolate (in that order) that were identified by MLST as belonging to the same subtype. The frequency of vaccine-selected capsular switch was defined as the weekly probability for a vaccine-type strain colonizing a vaccinated individual to be selected after it has switched its capsular type in order to evade vaccine-conferred immunity.

### Mathematical model of pneumococcal transmission in a vaccinated population

**Figure 1:**
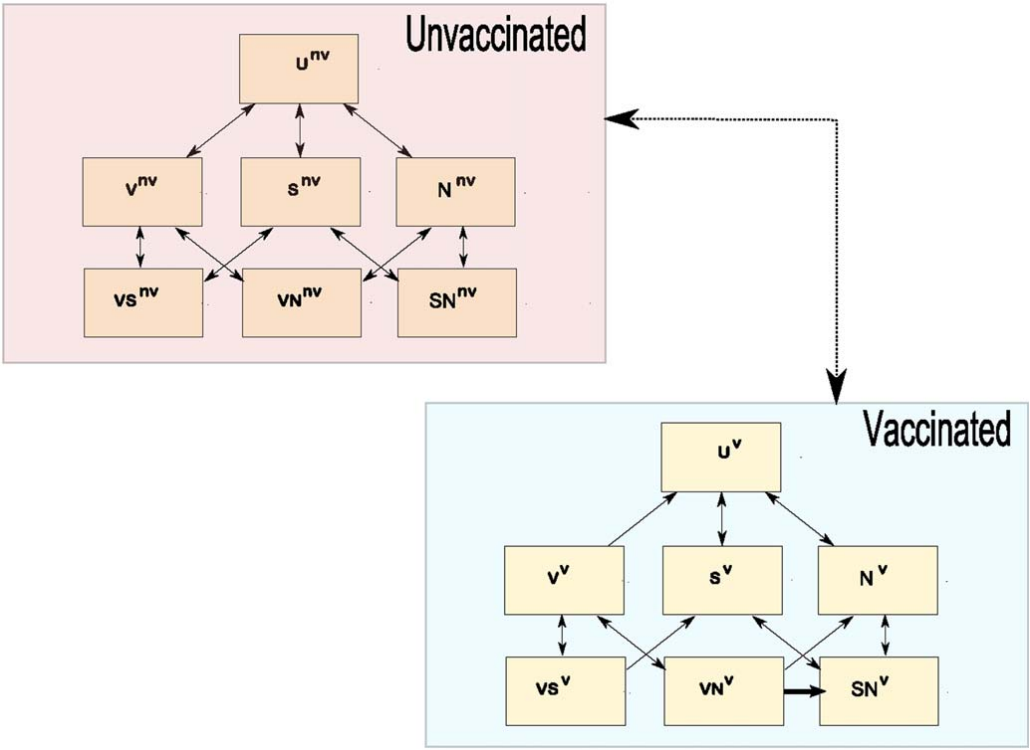
Model Structure within one age class. Individuals can be either uncolonized (U), colonized with vaccine serotypes (V), non-vaccine serotypes resulting from the capsular switch of a vaccine serotype (S) or wild-type non-vaccine serotypes (N). Dual colonization is allowed (VS, VN or SN). The model is structured into two age classes (children and adults). A portion of children are vaccinated, and vaccination immunity may last into adulthood. Vaccinated individuals can only acquire colonization with non-vaccine-type pneumococci. Switch events may occur in vaccinated individuals colonized with both vaccine and non-vaccine serotypes (VN) and are depicted by a bold arrow.

We developed a mathematical model of pneumococcal colonization in a partially vaccinated community (Figure 1). At any given time, individuals reside in a compartment according to their carrier status and to the characteristics of the pneumococcal strain they may carry. The model depends on several parameters, which are listed with their values in Table 1.

**Table 1:** Estimation of the switch frequency (weekly probability for a persistently carried pneumococcal strain to switch its capsule).

Study	Number of reported switches	Cumulated persistent carriage duration (weeks, estimated)	Estimated switch frequency (weeks^−1^)
(Barnes et al) [27]	1	121	8.2×10^−3^
(Sluijter et al)* [28]	1	169	5.9×10^−3^
(Meats et al) [19]	0	3852	0
(Bogaert et al) [29]	3	3068	9.7×10^−4^
*Random effects pooled estimate from the 4 studies * [*30*]			1.5×10^−3^ [4.6×10^−5^–4.8×10^−3^]

* Regarding the second study [28], it is to be noted that although the article provided the results of all positive samplings in chronological order for all children, it did not specify the corresponding sampling times. We therefore had to approximate the overall persistent colonization duration by supposing that all successive positive samplings occurred consecutively at a 1 month interval.

#### Capsular switch modeling

The model did not account for all switching, but only for “excess” vaccine-selected capsular switch, which may not be compensated. That is, we hypothesized that conjugate vaccination aided the selection of switched variants of the seven strains included in its formulation. The “natural” switch phenomenon, which affects all pneumococcal strains regardless of vaccination, needs not be explicitly modeled as switches to and from vaccine strains should compensate.

We investigated a frequency of vaccine-selected capsular switch in vaccinated individuals ranging from 0 (no vaccine-selected capsular switch) to 10^−2^/week.

#### Model structure

The model structure is depicted in Figure 1. The seven serotypes covered by the heptavalent conjugate vaccine (vaccine serotypes, namely serotypes 4, 6B, 9V, 14, 18C, 19F and 23F, representing 58% of all strains in Europe) are distinguished from other serotypes (non-vaccine serotypes, representing 42% of all strains). Non-vaccine serotypes deriving from the capsular transformation of vaccine serotypes are further separated from “wild-type” non-vaccine serotypes. The vaccine is supposed to be 100% effective against colonization with vaccine serotypes.

Individuals in the model reside in one of 7 carriage states at any given time: non carriers; carriers of vaccine serotypes (V); carriers of non-vaccine serotypes deriving from the capsular switch of vaccine serotypes (S); carriers of “wild-type” non-vaccine serotypes (N); and multiple carriers who are colonized with two different isolates (VS, VN and SN). Because the model also takes into account the vaccination status, meaning that individuals can be either vaccinated or not vaccinated, as well as two age classes (<2 years old and >2 years old), there are 28 compartments in all.

#### Colonization modeling

Both the first acquisition of a pneumococcal strain and the acquisition of a second strain occurred following an infectious contact with a colonized individual. The frequency of these infectious contacts depended on the age and colonization status of the host, as well as on the strain involved.

Observed colonization rates with vaccine and non-vaccine serotypes in the pre-vaccine era suggest a reduced fitness in the latter, which might be expressed by a lower transmissibility [20]. Here, we supposed a 0.98 ratio between the frequencies of infectious contacts involving non-vaccine and vaccine serotypes. Switched serotypes were supposed to have the same fitness as vaccine serotypes.

In order to model competition, the probability of acquisition of a particular serotype was reduced by 50% in hosts already carrying another serotype [7].

The frequencies of infectious contacts with vaccine strains in uncolonized children and adults were calibrated to reflect observed colonization rates in the pre-vaccine era (40% in children under 2 years old [21], 20% for “adults” over 2 years old [22]; 58% of vaccine serotypes).

The mean duration of colonization was supposed to be the same for all types of strains in the model. The duration of pneumococcal colonization was estimated from the literature at 13.8 weeks in children <2 years old, and at 4.4 weeks in adults [23].

#### Invasiveness modeling

We simplified our description by assuming that all vaccine serotypes were invasive, whereas only a portion of wild-type non-vaccine serotypes were. We also hypothesized that capsular switch did not affect invasiveness, implying that non-vaccine serotypes deriving from the capsular transformation of vaccine serotypes remained invasive.

#### From colonization to invasive disease

Invasive pneumococcal disease (IPD) incidence was presumed to occur in a portion of incident colonization cases with invasive strains.

A recently published study investigated serotype-specific invasiveness from longitudinal data on pneumococcal acquisition and infection in children <2 years old [23]. In this study, serotype-specific attack rates were defined as the ratio of the incidence of invasive pneumococcal disease to the incidence of acquisition and were calculated to determine the expected number of IPD cases for 100,000 acquisitions of each pneumococcal serotype. We used these serotype-specific attack rates to compute the attack rate of vaccine serotypes (13 IPD cases/100,000 acquisitions), as well as the attack rate of invasive non-vaccine serotypes (25 IPD cases/100,000 acquisitions) and the attack rate of non-invasive non-vaccine serotypes (3.25 IPD cases/100,000 acquisitions) in children. For adults, it is thought that immunological history induces a reduction in attack rates [20], [24]. We calculated adult attack rates by dividing children attack rates by 4.5, in order to reproduce the difference between observed incidences in children and adults in age-specific data on IPD [25].

We then determined the number of IPD cases/100,000 per annum from model predictions by multiplying the number of acquisitions of each strain type during a given year by the corresponding mean attack rate in each age class.

#### Sensitivity analysis

In order to evaluate the robustness of our results, we performed a sensitivity analysis of the model using the Latin hypercube sampling-partial rank correlation coefficients technique [26].

## Results

### Review on pneumococcal capsular switch

#### Selected studies (Table 2)

**Table 2:** Model parameters.

Parameter	Estimated value	Reference
Mean duration of pneumococcal colonization in children	13.8 weeks	[23]
Mean duration of pneumococcal colonization in adults	4.4 weeks	**
Rate of infectious contacts among children	0.4 weeks^−1^	**
Rate of infectious contacts between children and adults	0.25 weeks^−1^	**
Rate of infectious contacts among adults	0.26 weeks^−1^	**
Reduction of the acquisition probability in colonized individuals	50%	**
Non-vaccine isolates transmissibility-vaccine isolates transmissibility ratio (fitness parameter)	0.98	[36]
Attack rates in children (IPD cases per 100,000 acquisitions)		[23]
vaccine serotypes	13 IPD/100,000	
nonvaccine serotypes	6.5 IPD/100,000	
Attack rates in adults (IPD cases per 100,000 acquisitions)		[23], [25]
vaccine serotypes	3 IPD/100,000	
nonvaccine serotypes	1.5 IPD/100,000	
Mean duration of vaccine immunity after 2 years old	10 years	[48]
Vaccine coverage	0–100%	
Vaccine-selected capsular switch frequency	0–10^−2^ weeks^−1^	

** Value resulting from a calibration of the model in order to reproduce observed age-specific data on pneumococcal colonisation.

Our systematic search of Medline retrieved 147 original articles. Four epidemiological studies providing data on capsular switch were found consistent with our requirements [19], [27]–[29].

The first study [19] aimed specifically at estimating the frequency of serotype change. A birth cohort of a 100 infants was followed for 2 years. There was no evidence for any change of serotype due to capsular switch.

The second study [27] reported results from routine surveillance in a day care centre over 7 years; one capsular switch event was reported during an outbreak of colonization involving 14 children between May 1990 and December 1991.

In the third study [28], 19 children were monitored during the first 2 years of their life; one episode of capsular switch was reported.

The fourth and most recent study [29] was a randomized double-blind trial with the 7-valent conjugate pneumococcal vaccine involving 383 children who were followed for 6 months; 3 episodes of capsular switch were reported.

#### Estimation of the “natural” vaccine-independent switch frequency

Switch frequencies estimated from the 4 selected studies are summarized in Table 1 and vary between 0 and 8.10^−3^/week. We also computed a random effects pooled estimate of the switch frequency from all available data in the 4 studies, which included a total of 5 reported switches for an overall cumulated duration of persistent colonization of 5044 weeks (DerSimonian and Laird meta-analysis [30]). The estimated frequency was 1.5×10^−3^/week (95% confidence interval, [4.6×10^−5^/week–4.8×10^−3^/week]).

#### Investigation of vaccine-selected “excess” capsular switch

Only one of the 4 selected studies provided data on the vaccination status of colonized individuals [29]. Two more studies of the impact of conjugate vaccination on pneumococcal colonization were identified [31], [32]; these 2 studies looked at capsular switch but did not provide full individual data.

Neither of these studies provided any major evidence for an increased frequency of strains with suspected capsular changes among pneumococci isolated from the nasopharyngeal samples of vaccinees. In the last identified study [32], possible capsular switching from a non-vaccine serotype toward a vaccine serotype was detected once among 197 isolates collected over 1 year in vaccinated children.

As a whole, the available data was too scarce to allow any conclusions to be drawn regarding the possibility of an increased switch frequency due to the selective immunological pressure of vaccination.

### Mathematical model: validation and predictions

**Figure 2:**
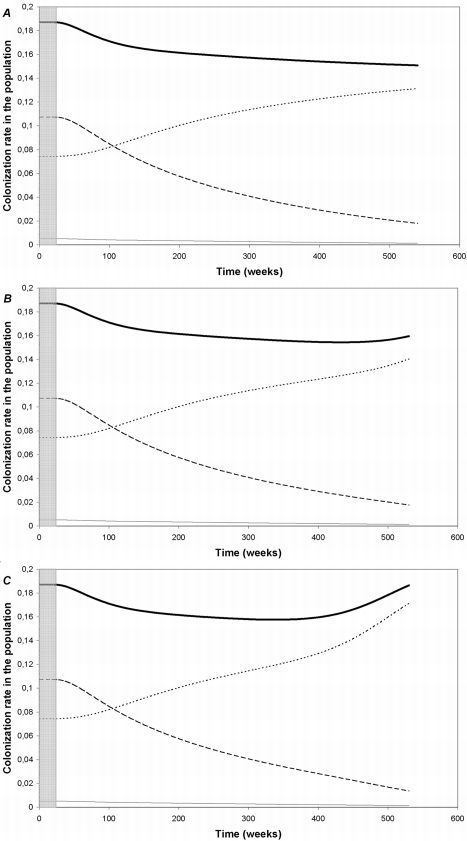
Time changes in the pneumococcal population in the post-vaccine era: global colonization rate (bold line), colonization with vaccine serotypes (dashed line), non-vaccine serotypes (dotted lines), and dual colonization with vaccine and non-vaccine serotypes (full line), a) with a frequency of vaccine-selected capsular switch _←_ = 0, b) with a frequency of vaccine-selected capsular switch _←_ = 10^−4^/week, and c) with a frequency of vaccine-selected capsular switch _←_ = 10^−3^/week.

**Figure 3:**
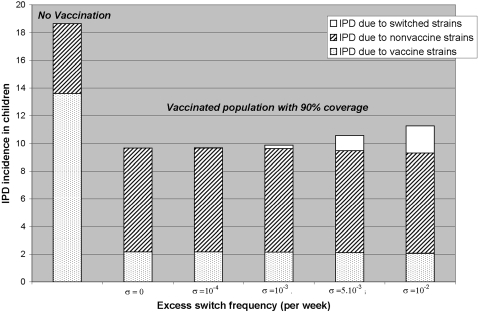
Invasive Pneumococcal Disease (IPD) incidence in children <2 years old (per 100,000 children, per year) 5 years after the introduction of a conjugate vaccine, as a function of the frequency of capsular switch selected in vaccinated individuals, for a 90% vaccination coverage. The origin of the cases (vaccine strains, non-vaccine strains, switched strains) is specified, and the expected incidence without vaccination is also depicted as a reference.

We simulated the introduction of the heptavalent conjugate vaccine with 90% coverage, under various scenarios regarding the actual frequency of vaccine-selected capsular switch in vaccinated individuals. Figure 2 depicts time changes in the colonizing pneumococcal population in the post-vaccine era, with a frequency of vaccine-selected capsular switch fixed at 0 (Figure 2a), 10^−4^/week (Figure 2b) and 10^−3^/week (Figure 2c). Figure 3 provides the yearly incidence of IPD in children 5 years after the introduction of the vaccine for vaccine-selected capsular switch frequencies between 0 and 10^−2^/week; the pre-vaccination incidence is also presented as a reference.

#### “Long-term” pneumococcal population

Following the introduction of the vaccine, colonization with vaccine-type isolates decreased while colonization with non-vaccine-type isolates increased (Figure 2). Both the speed and the extent of this replacement increased with the frequency of vaccine-selected capsular switch. For a 10^−3^/sem frequency of vaccine-selected capsular switch, the overall prevalence of carriage in the population had regained its pre-vaccine level after 10 years of simulated vaccination (Figure 2c).

Switched strains constituted more than 30% of all invasive strains for vaccine-selected capsular switch frequencies over 10^−4^/week. However, non-invasive strains still represented at least half of all pneumococcal strains, even for high selected switch frequencies in vaccinated individuals (data not shown).

Simulations with a less extensive vaccination coverage led to similar results provided the coverage remained over 40%.

#### Comparison of model predictions with observed data

In most European countries, the reported incidence of IPD in the pre-vaccination era was between 10 and 24 cases/100,000 per annum among children [25], [33]. This is consistent with the 18.7 IPD cases/100,000 children predicted by the model in the absence of vaccination (Figure 3).

After 5 years of vaccination, the predicted annual incidence was reduced by more than 40% in children (Figure 3), and more than 30% in the general population (data not shown). This is consistent with observed reductions in European vaccinated populations [34], [35].

#### Predictions on the impact of capsular switch

Without any vaccination-selected switch, the expected IPD incidence was reduced by 48% after 5 years of vaccination. For switch frequencies up to 10^−2^/week, this reduction remained significant at over 40% (Figure 3).

However, vaccine-selected switch in vaccinated individuals led to excess IPD cases. Table 3 provides the expected number of supplemental IPD cases in children, cumulated over the 10 years following the introduction of conjugate vaccination. A high vaccine-selected switch frequency (over 7,5.10^−3^/week) induced 3 excess IPD cases per 100,000 children over these 10 years.

**Table 3:** IPD cases due to capsular switch. Number of IPD cases due to switched pneumococcal strains, per 100,000 children under 2 years old, cumulated over the 10 years following the introduction of conjugate vaccination, as a function of the frequency of capsular switch selected in vaccinated individuals.

Vaccine-selected switch frequency (weeks^−1^)	Number of IPD cases (for 100,000 children) due to switched strains, cumulated over 10 years
0–2×10^−4^	0
3×10^−4^–1.6×10^−3^	1
1.7×10^−3^–5.8×10^−3^	2
5.9×10^−3^–10^−2^	3

Vaccination coverage is fixed at 90%.

#### Sensitivity analysis

The results of the sensitivity analysis are given in Table 4. The most critical parameters for predicting the incidence of IPD in the post-vaccine era were the duration of colonization in adults and the rate of infectious contacts between adults. Other important parameters were the rate of infectious contacts between children and adults, as well as the attack rate of non-vaccine serotypes.

**Table 4:** Sensitivity analysis of the incidence of IPD 5 years after the introduction of conjugate vaccination. Vaccination coverage is fixed at 90% and the frequency of vaccine-selected capsular switch is fixed at 10^−3^/week. Critical parameters are in bold.

Parameter	PRCC^a^
Mean duration of pneumococcal colonization in adults	**0.925**
Mean duration of pneumococcal colonization in children	0.223
Rate of infectious contacts among children	0.048
Rate of infectious contacts between children and adults	**0.469**
Rate of infectious contacts among adults	**0.896**
Reduction of the acquisition probability in colonized individuals	0.265
Non-vaccine isolates transmissibility-vaccine isolates transmissibility ratio (fitness parameter)	0.071
Attack rate of vaccine serotypes	0.134
Attack rate of non-vaccine serotypes	**0.369**
Mean duration of vaccine immunity after 2 years old	0.060

a Partial rank correlation coefficients (PRCC) indicate the degree of monotonicity between a specific input variable and a particular outcome variable. The sign of the PRCC indicates the qualitative relationship between input and output variables. The magnitude indicates the importance of uncertainty in estimating the value of the input variable in contributing to the imprecision in predicting the value of the outcome variable.

## Discussion

Combining literature review and mathematical modelling, we investigated the potential impact of capsule switching on the efficacy of conjugate pneumococcal vaccination. Although there was not enough statistical power to quantify precisely the vaccine-selected capsular switch frequency among vaccinated individuals, we found that this phenomenon should not lessen significantly the reduction in IPD incidence induced by the vaccine. This is in part due to competition between switched isolates and other non-vaccine isolates in vaccinated populations.

### Discussion of the switch frequency estimation

Estimating the switch frequency from published studies only should be challenged, as available data are scarce and exhibit several limitations, such as being restricted to antibiotic-resistant populations, specific age groups or invasive infections [18].

Because the data was so scarce, we could only estimate the switch frequency with a wide confidence interval. Moreover, it is possible that this frequency depends on the pneumococcal serotype or on characteristics of the human host (such as age), but we were unable to investigate this hypothesis.

Finally, it could be argued that there is no direct proof that the serotype changes reported in the studies we selected are indeed due to capsular switch, as many events can go unobserved between two consecutive samplings. This is all the more true when samplings occur many months apart. For instance, in the study by Bogaert and colleagues [29], it is possible that the observed consecutive colonization at a 6-month interval with different serotype variants of closely related strains merely suggests the recruitment of a second isolate with identical genotype. Conversely, it is also possible that some capsular switch events may have gone undetected in this study. It would be interesting to use a data augmentation technique such as Markov Chain Monte Carlo on the data from these studies in order to obtain more robust estimates of the switch frequency [36].

### Discussion of model hypotheses

#### Vaccine-independent capsular switch vs. vaccine-selected capsular switch

We hypothesized that in the absence of vaccine pressure, switches to and from vaccine strains should be balanced. This allowed us not to take into account vaccine-independent capsular switch in our model. Although it seems possible that the underlying genetic nature of different switches may have specific directionality, our balance hypothesis is supported by the fact that, in a given location, the frequency distribution of particular pneumococcal serotypes or serogroups in IPD has remained nearly constant over several decades before conjugate vaccination [20].

In vaccinated individuals however, non-vaccine isolates which result from the natural switch of a vaccine isolate may persist under selective pressure due to vaccination, whereas vaccine isolates will not. Therefore, we included the possibility for vaccine-selected capsular switch in vaccinated individuals in our model.

#### Range of values for the vaccine-selected switch frequency

The emergence of new clones expressing non-vaccine serotypes has been observed in countries where the vaccine was widely used [14]. However, although this raises suspicions that conjugate vaccination might increase the frequency of capsule switching from vaccine-covered types, quantification is not yet possible from epidemiological data. Here, we supposed that vaccination could not do much more than double the overall “natural” propensity of pneumococci to switch their capsule. Based on our estimation of the frequency of natural capsular switch between 5.10^−5^/week and 5.10^−3^/week, this led us to investigate vaccine-selected switch frequencies in vaccinated individuals between 0 and 10^−2^/week.

In order to be as conservative as possible, we also investigated vaccine-selected switch frequencies up to 0.1/week, although we chose not to present this data in this article. For such high frequencies, we expect switched isolated to be responsible for nearly half of IPD cases 5 years after the introduction of the vaccine. However, because this increase in switched isolates should for the most part occur as replacement of non-vaccine isolates, rather than add to the overall colonization, the impact on IPD incidence may remain limited. For instance, vaccine-selected capsular switch should not lead to more than 5 excess IPD cases per 100,000 children over the 10 years following the introduction of conjugate vaccination.

#### Capsular type and pathogenicity

A major model hypothesis is that capsular switch does not affect the pathogenicity of a given pneumococcal strain, implying that a highly virulent vaccine strain would still be as virulent if it switched its capsular type to express a non-vaccine serotype. However, this is a widely debated issue, as earlier studies have shown complicated interactions between capsular type and other genes in determining pneumococcal virulence.

The capsule has long been identified as a virulence factor by virtue of its antiphagocytic activity, and acapsular mutants are known to be avirulent [37]. Several molecular epidemiology studies have shown a clear association between the pneumococcal capsule and the ability of pneumococci to cause invasive disease [38], [39]. However, in two recent studies, isolates belonging to the same clone but with different capsules because of serotype switch were found to have the same disease potential [24], [40].

According to several experimental studies based on the construction of chimeric pneumococcal mutants with a switched capsular type, the capsule type is not the only determinant of pathogenicity [41], [42]. Hence the virulence of a switched strain may not be predictable from that of the virulence of the original strain. Moreover, other genetic factors might be involved in colonization and in non-invasive pneumococcal disease [43].

In this study, we aimed at evaluating the potential impact of capsular switch on the long-term effectiveness of current pneumococcal conjugate vaccines, based on the suspicion that the constant usage of these vaccines may aid the expansion of serotype switch variants. In that context, our hypothesis that switched vaccine-type pneumococcal strains retained their higher virulence despite expressing non-vaccine serotypes constituted a worst-case scenario.

#### Multiple colonization and competition

Colonization studies show that many people carry more than one pneumococcal type at the same time, although multiple colonization is less frequent than would be expected if each serotype circulated independently of the others [44]. Because the most probable mechanism for vaccine-selected capsular switching involves multiple colonization of a vaccinated host, we allowed for dual colonization in our model, but we also hypothesized that there was some level of competition. We modelled competition by a reduction in the probability of acquisition of a second strain in an already colonized host [7]. Although interference between pneumococcal strains is documented [45], there was little data to estimate this competition parameter. Based on the sensitivity analysis where we investigated reductions in acquisition probability ranging from 25% to 75%, this parameter does not appear to be the most critical for predicting IPD incidence in the post-vaccine era.

#### Fitness

In the pre-vaccine era, the portion of pneumococcal colonization attributable to vaccine serotypes seems to have remained stable over time at a level >50% in various parts of the world, although it varied geographically. In a context of multiple colonization, this strongly suggests that vaccine serotypes have an increased fitness for colonization. This fitness may be expressed either as increased carriage duration or as higher transmissibility. However, in an earlier study based on longitudinal data, we found little to no differences in the mean duration of carriage and transmission rates of vaccine serotypes and nonvaccine serotypes groups [36]. Here, we included in the model a slightly reduced transmissibility for non-vaccine serotypes; even with identical carriage durations, this allowed us to reproduce the observed differences in prevalence before the introduction of the vaccine.

### Discussion of model predictions

Because we evaluated the invasive attack rates of pneumococci from European data [23], we used European data on IPD incidence to validate model predictions. This incidence is significantly inferior to that reported in the United States, as a 4 to 7-fold difference has been reported between the United States and Europe in the pre-vaccination era [25], [33]. This may arise from different medical practices between countries, such as admission thresholds and blood culture rates [25]. Model parameters could easily be adjusted to reflect US IPD incidence, provided data on serotype-specific attack rates in the US became available.

According to our predictions, the reduction in IPD incidence after 5 years of vaccination should only be around 40–50% in children, whereas observations in the United States showed a 69% reduction in children under 2 years old after 3 years of vaccination [2]. Reasons for this apparent discrepancy are twofold. First, again, we focused on the European rather than US context; this means that the initial serotype coverage of the heptavalent vaccine was noticeably less favourable (58% of carried serotypes instead of approximately 80%). Second, as evidenced in previous work [6], our model predicts the replacement of vaccine serotypes by non-vaccine serotypes. Despite differing attack rates, this should be expected to lessen the impact of vaccination on IPD incidence within a five-year timeframe [8]. Recent data showing a re-increase in non-vaccine IPD incidence in the US support these predictions [5], [46], [47].

### Conclusions

Despite the significant short-term impact of conjugate pneumococcal vaccination reported in the US, it has been predicted from mathematical models that non-vaccine serotypes may come to replace vaccine serotypes, with implications for the long-term effectiveness of the vaccines. This is supported by recent data from various countries [47]. However, suspicions that pneumococci expressing vaccine serotypes may be incited to switch their capsule, thereby evading vaccine immunity while retaining their invasiveness and their high levels of antibiotic resistance, may be even more worrying [12]. Indeed, this could lead to a re-increase in highly resistant IPD in vaccinated populations.

The new conjugate vaccines with increased valence which should be available in years to come might–at least in the short term-help lessen the extent and speed of serotype replacement [6]. On the other hand, the potential impact of vaccine-selected capsular switch could only increase with the number of serotypes covered by the vaccine.

Here, we show that, based on the limited data available, the extent of this phenomenon should remain quite limited. This is in agreement with recent data from the US [32]. Nevertheless, this study stresses the major importance of collecting surveillance data in vaccinated populations which will allow for the detection of any switch event from vaccine to non-vaccine serotypes, as well as for the quantification of the frequency of these events. This should also be kept in mind when designing and analysing clinical trials for new pneumococcal vaccines with higher valence.
